# Substantial differences in perception of disease severity between post COVID-19 patients, internists, and psychiatrists or psychologists: the Health Perception Gap and its clinical implications

**DOI:** 10.1007/s00406-023-01700-z

**Published:** 2023-11-13

**Authors:** Michael Ruzicka, Gerardo Jesus Ibarra Fonseca, Simone Sachenbacher, Fides Heimkes, Fabienne Grosse-Wentrup, Nora Wunderlich, Christopher Benesch, Anna Pernpruner, Elisabeth Valdinoci, Mike Rueb, Aline Olivia Uebleis, Susanne Karch, Johannes Bogner, Julia Mayerle, Michael von Bergwelt-Baildon, Marion Subklewe, Bernhard Heindl, Hans Christian Stubbe, Kristina Adorjan

**Affiliations:** 1 Department of Medicine III, Ludwig Maximilian University (LMU) University Hospital, LMU Munich, Marchioninistrasse 15, 81377 Munich, Germany; 2 Department of Medicine IV, LMU University Hospital, LMU Munich, Munich, Germany; 3 Department of Psychiatry and Psychotherapy, LMU University Hospital, LMU Munich, Munich, Germany; 4 Department of Medicine II, LMU University Hospital, LMU Munich, Munich, Germany; 5 German Center for Infection Research, Partner Site Munich, Munich, Germany; 6 Stabstelle Strategische Unternehmenssteuerung, LMU Munich, Munich, Germany

**Keywords:** Post COVID-19 syndrome, Long COVID, Disease perception, Mental health, Psychosomatic, Depression

## Abstract

Patient-reported outcome measures (PROMs) such as the Numeric Pain Rating Scale (NPRS) or Likert scales addressing various domains of health are important tools to assess disease severity in Post COVID-19 (PC) patients. By design, they are subjective in nature and prone to bias. Our findings reveal substantial differences in the perception of disease severity between patients (PAT), their attending internists (INT) and psychiatrists/psychologists (PSY). Patients rated almost all aspects of their health worse than INT or PSY. Most of the differences were statistically highly significant. The presence of fatigue and mood disorders correlated negatively with health perception. The physical health section of the WHO Quality of Life Assessment (WHOQoL-BREF) and Karnofsky index correlated positively with overall and mental health ratings by PAT and INT. Health ratings by neither PAT, PSY nor INT were associated with the number of abnormal findings in diagnostic procedures. This study highlights how strongly perceptions of disease severity diverge between PC patients and attending medical staff. Imprecise communication, different experiences regarding health and disease, and confounding psychological factors may explain these observations. Discrepancies in disease perception threaten patient-physician relationships and pose strong confounders in clinical studies. Established scores (e.g., WHOQoL-BREF, Karnofsky index) may represent an approach to overcome these discrepancies. Physicians and psychologists noting harsh differences between a patient’s and their own perception of the patient’s health should apply screening tools for mood disorders (i.e., PHQ-9, WHOQoL-BREF), psychosomatic symptom burden (SSD-12, FCV-19) and consider further psychological evaluation. An interdisciplinary approach to PC patients remains imperative. *Trial Registration Number & Date of Registration*: DRKS00030974, 22 Dec 2022, retrospectively registered.

## Introduction

Following several pandemic waves of the severe acute respiratory syndrome coronavirus type 2 (SARS-CoV-2), an increasing number of patients report long lasting, post-acute sequelae of Coronavirus Disease 2019 (COVID-19). Among other labels, these are commonly referred to as the Post COVID-19 (PC) syndrome. PC manifestations include a broad variety of possible symptoms of predominantly pulmonary, cardiologic and neuropsychiatric nature [1, 2]. By definition of the World Health Organization (WHO), a PC condition may be diagnosed if symptoms lasting for a minimum of 2 months newly develop or persist 3 months after the initial SARS-CoV-2 infection with no other causal explanation [3]. While the prevalence of post-acute disease manifestations varies greatly across the published literature, the WHO estimates that PC conditions develop in 10–20% of COVID-19 cases [3].

Due to its heterogeneous nature, frequent multiple organ involvement and high prevalence, the PC syndrome poses an important challenge to health care providers across the globe. An interdisciplinary approach is crucial to meet the many facets of the condition. Often, a broad range of diagnostic tools and assessments is required, including physical examination, laboratory and/or endocrinologic tests, pulmonary function assessments, cardiac assessments such as electrocardiograms (ECG) and transthoracic echocardiography (TTE), radiological approaches (i.e. pulmonary or brain imaging), neuropsychiatric evaluation, cognitive testing and many more [4–7]. Yet, even extensive diagnostic workups fail to identify satisfying points of therapeutic action in a relevant portion of patients with persistent physical and/or psychological distress. Despite ongoing research efforts, the pathogenesis behind the PC syndrome remains elusive and no causal treatments have been identified at the time of writing.

The complexity of the disease and the broad spectrum of suggested diagnostic approaches often impedes prompt beginning of efficient treatment or measures of rehabilitation. Inaccurate communication between patients and health care professionals leads to improper or redundant diagnostic measures, hinders identification of points of therapeutic action, and can hamper progress even further. Beyond, PC patients often articulate frustration due to feeling misunderstood or not taken seriously by attending physicians. Lastly, at the time of writing, no biomarkers have yet been identified to confirm the presence or objectively monitor the course of a PC condition. Consequently, patient-reported outcome measures (PROMs) are frequently used as an alternative to this end. Due to their subjective nature, they are prone to bias. Thorough validation of the various PROMs available in the context of the PC condition is desirable as they often pose the foundation for physician–patient communication, therapeutic decisions, or the evaluation of therapeutic progress in clinical and investigational/study settings.

To tackle the issues mentioned above, we assessed differences in disease perception between PC patients (PAT), their attending internists (INT), and psychologists/psychiatrists (PSY). We investigated underlying disease and circumstantial factors that confound disease perception and lead to miscommunication between patients and healthcare providers. Further, we tested various PROMs and other established clinical scores to identify the most fitting ones to overcome miscommunication and accurately assess a PC patients’ condition.

## Patients and methods

The Post-COVID-Care Study (DRKS00030974) is a prospective cohort study designed to characterize the nature and course of the PC syndrome. Patients who were treated in our Post-COVID^LMU^ outpatient department were enrolled in the study if the criteria for a PC condition as defined by the WHO [3] were met and if the initial COVID-19 diagnosis was confirmed by SARS-CoV-2 PCR testing. All patients provided written informed consent before study inclusion. For this work, we performed a cross-sectional analysis of disease perceptions by patients themselves and attending health care workers. To this end, patients (PAT), psychologists/psychiatrists (PSY), and internists (INT) rated the patients’ health status in the following categories:

1. “Overall health” (OH)
2. “Mental health” (MH)
3. “Physical health” (PH)
4. Pain

The patients’ OH, MH and PH was rated in analogy to the Patient-Reported Outcomes Measurement Information System—Global Health 10 (PROMIS-10) using a Likert scale composed of 5 grades: “excellent” (5), “good” (4), “fair” (3), “poor” (2) and “very poor” (1). Pain was assessed using the Numeric Pain Rating Scale (NPRS) ranging from no pain (0), very light (1) to extreme pain (10). Patients answered the WHO Quality of Life Assessment (WHOQoL-BREF; [8]), 9-item Patient Health Questionnaire (PHQ-9; [9, 10]) and Fatigue Severity Scale (FSS; [11, 12]). WHOQoL-BREF assesses the patients’ quality of life on a 0–100 scale in 4 various domains: physical and psychological health, social relationships, and environment. Higher values reflect a better quality of life, and results were interpreted based on the following reference values established by Hawthorne et al.: 73.5 [standard deviation (SD) = 18.1] for physical health, 70.6 (SD = 14.0) for psychological health, 71.5 (SD = 18.2) for social relationships and 75.1 (SD = 13.0) for the environment domain [13]. The PHQ-9 offers a possibility to screen for and grade depressive disorders by severity on a 0–27 scale, and the FSS provides a tool to quantify fatigue on a 0–63 scale with a cutoff for pathological values at ≥ 36 points.

All clinical data were documented by means of the Lightweight Clinical Data Acquisition and Management Software for Clinical Research (LCARS, LMU University Hospital, Germany). PAT, INT and PSY reported their findings via the progressive web app module of LCARS (LCARS-M). INT and PSY clinically assessed the patients simultaneously, and the findings by INT, PSY and PAT were documented immediately after the clinical assessments. Their reports were independent and blinded regarding the reports by others. Clinical data relevant to this study were extracted from the LCARS database.

Statistical tests were conducted using R Studio version 4.2.1. Numeric variables are depicted as median values (Mdn) followed by the respective interquartile ranges (IQR) in square brackets. Categorical variables are given as absolute counts with respective percentages in round brackets. 95% confidence intervals (CI) are depicted separately. Statistical significance between groups was calculated with a two-sided Kruskal–Wallis test for numeric variables. Pearson’s Chi-squared test was used to assess statistical differences between categorical data. Correlations were tested with the Spearman correlation. *P* values were adjusted for multiple comparisons by applying the Benjamini–Hochberg procedure if indicated and were considered as statistically significant at values < 0.05.

## Results

### Patient characteristics and PROMs

A total of 315 patients from our Post-COVID^LMU^ outpatient department with a likely or confirmed PC syndrome were enrolled in our study. OH, MH, PH and pain were rated by the patients themselves, their treating INT, and PSY independently. This assessment of disease perception was completed in 71 of the 315 cases. Of these 71 patients, the majority was female (56% female vs 44% male; Table 1). The Mdn age at inclusion was 39 [30, 51] years. Most patients reported to be on sick leave (57%) due to the PC condition. The inability to work was also reflected by a Mdn Karnofsky index of 70% [70, 80]. In 88.7% of the cases the initial SARS-CoV-2 infection was non-severe and only 4.2% of patients were hospitalized. At the time point of inclusion, 17 (24%) of the patients had a history of psychiatric conditions with depression (13%) and chronic fatigue syndrome (8.5%) being the most common (Table 1). In 15 (21%) patients, psychiatric conditions were known prior to SARS-CoV-2 infection. 5 (7%) patients were diagnosed with a psychiatric condition after SARS-CoV-2 infection. A Mdn of 4 [3, 6] preexisting somatic conditions was found with 54 (76%) of the patients having a history of at least one somatic disease. Bronchial asthma (14%) and primary hypertension (13%) were the most prevalent somatic diagnoses (Table 1). 45 (63%) patients had known somatic conditions prior to SARS-CoV-2 infection, 21 (30%) acquired new somatic diagnoses after infection.

**Table 1:** Patient characteristics and PROMs

	Number of patients (%)/[IQR] (*n*_total_ = 71)
Sex at birth	
Female	40 (56%)
Male	31 (44%)
Age at inclusion (years)	39 [30, 51]
Karnofsky index	70 [70, 80]
On sick leave	
Yes	39 (57%)
No	23 (33%)
Unknown	7 (10%)
Course of initial SARS-CoV-2 infection	
Outpatient	68 (95.8%)
Hospitalized	3 (4.2%)
Initial SARS-CoV-2 infection—WHO disease severity^a^	
Asymptomatic	1 (1.4%)
Non-severe	63 (88.7%)
Severe	3 (4.2%)
Critical	3 (4.2%)
Unknown	1 (1.4%)
Preexisting (neuro-)psychiatric condition	17 (24%)
Depression	9 (13%)
Chronic fatigue syndrome	6 (8.5%)
Cognitive impairment	4 (5.6%)
Preexisting somatic condition	54 (76%)
Primary (essential) hypertension	9 (13%)
Myocarditis	7 (9.9%)
Bronchial asthma	10 (14%)
Hypercholesterinemia	4 (5.6%)
Pulmonary embolism	4 (5.6%)
Hypothyroidism	3 (4.2%)
PROMs	
WHOQoL-BREF—physical health (points)	44 [31, 54]
WHOQoL-BREF psychological health (points)	58 [46, 67]
WHOQoL-BREF social relationship (points)	75 [58, 75]
WHOQoL-BREF environment (points)	72 [66, 78]
PHQ-9 (points)	10.0 [6.0, 14.0]
FSS (score)	57 [48, 60]

*PROMs* Patient-reported outcome measures

^a^Disease severity was assessed by analogy with the WHO severity definitions of COVID-19 [14], adding the categories “asymptomatic” and “unknown”

Results of the physical health domain of the WHOQoL-BREF showed a considerably low Mdn value of 44 [31, 54] points (Table 1) well below the reference margin (for reference values, see “Patients and Methods”). Likewise, the psychological health section revealed an impaired Mdn value of 58 [46, 67] points, while the social relationship (Mdn points 75 [58, 75]) and the environment section (Mdn points 72 [66, 78]) did not appear to be negatively affected. The Mdn PHQ-9 score was 10.0 [6.0, 14.0], reflecting moderate depression severity (based on Kroenke et al. [10]). A Mdn FSS of 57 [48, 60] indicated a high degree of clinically relevant fatigue.

### Differences in disease and pain quantification between PAT, INT and PSY

**Fig. 1:**
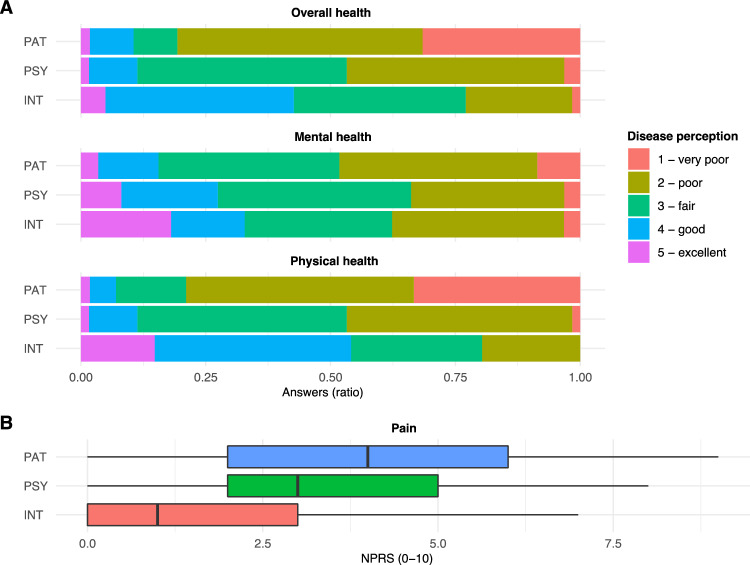
Detailed results of health assessments by PAT, INT and PSY. Patients, INT and PSY assessed PC patients’ (*n* = 71) health on Likert scales (**A**) or pain using the NPRS (**B**) as described in Patients and Methods. **A** The results are shown as ratios of all answers. **B** Bold vertical lines indicate median values; the colored horizontal bars resemble IQRs

Comparing health ratings by patients themselves, their attending INT and PSY, we found statistically significant differences with respect to OH (*p* < 0.001; Table 2), PH (*p* < 0.001) and pain assessments (*p* < 0.001) between the three groups. For example, 33% of patients rated their physical health as very poor (1), while PSY found this to apply in only 1.6% and INT in 0% of the cases (Fig. 1). In contrast, the ratings of the patients’ MH did not differ to a statistically significant extent (*p* = 0.063; Table 2).

**Table 2:** Comparison of disease perceptions by PAT, INT and PSY

Variable	INT (*n* = 71)	95% CI	PSY (*n* = 71)	95% CI	PAT (*n* = 71)	95% CI	*p* value^a^
Overall health	3.00 [3.00, 4.00]	3.0, 3.5	3.00 [2.00, 3.00]	2.4, 2.8	2.00 [1.00, 2.00]	1.7, 2.3	< 0.001
Mental health	3.00 [2.00, 4.00]	2.8, 3.4	3.00 [2.00, 4.00]	2.7, 3.2	3.00 [2.00, 3.00]	2.4, 2.9	0.063
Physical health	4.00 [3.00, 4.00]	3.2, 3.7	3.00 [2.00, 3.00]	2.5, 2.8	2.00 [1.00, 2.00]	1.7, 2.2	< 0.001
Pain	1.00 [0.00, 3.00]	1.1, 2.1	3.00 [2.00, 5.00]	2.7, 3.9	4.00 [2.00, 6.00]	3.1, 4.5	< 0.001

^a^Kruskal–Wallis rank sum test; *p* values adjusted by Benjamini–Hochberg method

Figure 1 gives detailed insight into the health assessments by the three groups. Respective subgroup analyses of Table 2 are depicted in Table 3A–C. The latter demonstrate that the evaluations of patients’ OH (*p* < 0.001) and PH (*p* < 0.001) differed significantly between PSY and PAT, while the ratings of patients’ MH and degree of pain [*p* values for both = not significant (n.s.)] appeared to be more in line between those two groups (Table 3A). In contrast, comparison of the INT and PAT health evaluations showed statistically significant differences in all fields but MH (*p* < 0.001 for OH, PH and pain; *p* = n.s. for MH; Table 3B). Lastly, when comparing INT and PSY health assessments of the patients, MH evaluations seemed to tightly align (*p* = n.s.), while OH (*p* < 0.001), PH (*p* < 0.001) and pain (*p* < 0.001) assessments did also differ significantly (Table 3C).

**Table 3:** Subgroup analysis of disease perceptions

A. PSY vs PAT					
Variable	PSY (*n* = 71)	95% CI	PAT (*n* = 71)	95% CI	*p* value^a^
Overall health	3.00 [2.00, 3.00]	2.4, 2.8	2.00 [1.00, 2.00]	1.7, 2.3	< 0.001
Mental health	3.00 [2.00, 4.00]	2.7, 3.2	3.00 [2.00, 3.00]	2.4, 2.9	*n.s.*
Physical health	3.00 [2.00, 3.00]	2.5, 2.8	2.00 [1.00, 2.00]	1.7, 2.2	< 0.001
Pain	3.00 [2.00, 5.00]	2.7, 3.9	4.00 [2.00, 6.00]	3.1, 4.5	*n.s.*

B. INT vs PAT					
Variable	INT (*n* = 71)	95% CI	PAT (*n* = 71)	95% CI	*p* value^a^
Overall health	3.00 [3.00, 4.00]	3.0, 3.5	2.00 [1.00, 2.00]	1.7, 2.3	< 0.001
Mental health	3.00 [2.00, 4.00]	2.8, 3.4	3.00 [2.00, 3.00]	2.4, 2.9	*n.s.*
Physical health	4.00 [3.00, 4.00]	3.2, 3.7	2.00 [1.00, 2.00]	1.7, 2.2	< 0.001
Pain	1.00 [0.00, 3.00]	1.1, 2.1	4.00 [2.00, 6.00]	3.1, 4.5	< 0.001

C. INT vs PSY					
Variable	INT (*n* = 71)	95% CI	PSY (*n* = 71)	95% CI	*p* value^a^
Overall health	3.00 [3.00, 4.00]	3.0, 3.5	3.00 [2.00, 3.00]	2.4, 2.8	< 0.001
Mental health	3.00 [2.00, 4.00]	2.8, 3.4	3.00 [2.00, 4.00]	2.7, 3.2	*n.s.*
Physical health	4.00 [3.00, 4.00]	3.2, 3.7	3.00 [2.00, 3.00]	2.5, 2.8	< 0.001
Pain	1.00 [0.00, 3.00]	1.1, 2.1	3.00 [2.00, 5.00]	2.7, 3.9	< 0.001

^a^Kruskal–Wallis rank sum test; *p* values adjusted by Benjamini–Hochberg method

### Correlation analysis of disease perceptions between PAT, INT and PSY

**Fig. 2:**
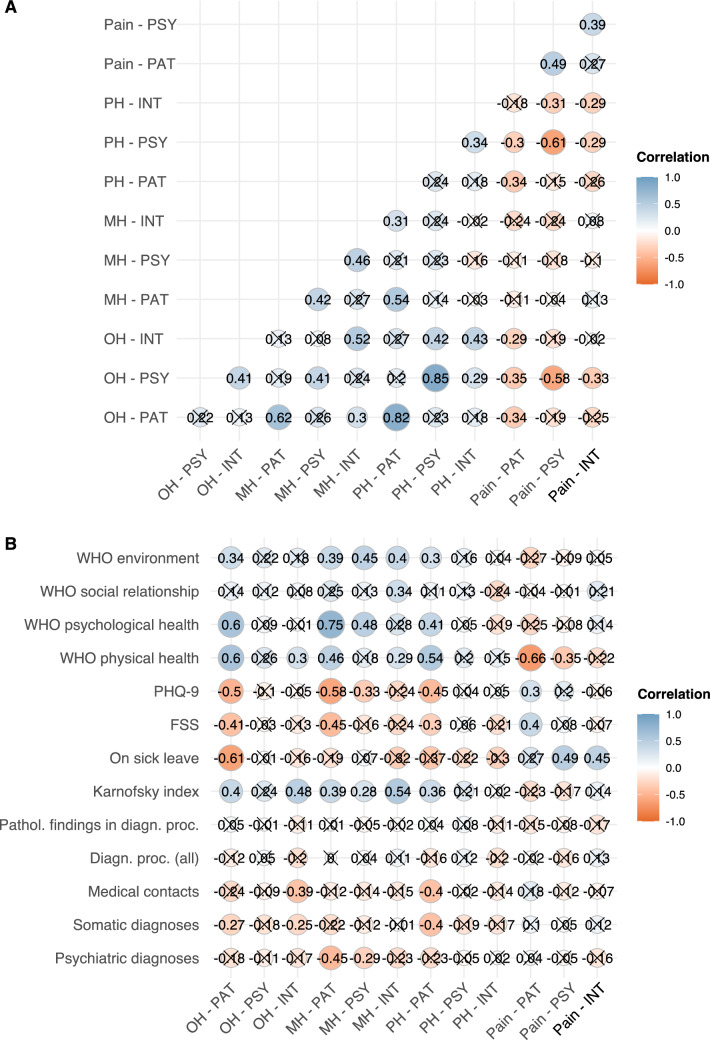
Correlation analyses of disease perceptions by PAT, INT, PSY between the groups, with other PROMs and objectifiable findings. Correlation matrices of disease perceptions by INT, PSY and PAT (*n* = 71). Correlations between the three groups and between disease perceptions and other PROMs, results of diagnostic procedures (number of all procedures or number of pathological outcomes) and other objectifiable findings were tested using the Spearman correlation. WHO environment, social relationship, psychological health and physical health refer to the respective sections of the WHOQoL-BREF. The figure shows Spearman’s *R*. Non-significant correlations (*p* value ≥ 0.050) are crossed out

We observed marked differences in medians of health estimates between the three groups. To investigate if the health ratings correlated despite differences in medians, we created a correlations matrix for all health ratings (Fig. 2A).

Despite statistically significant differences in the quantitative grading of patients’ OH and PH, but not MH and pain, between PSY and PAT (Table 3A), the correlation analysis revealed a positive correlation for both MH and pain (Spearman’s *R* for MH: 0.42, *p* < 0.001; Spearman’s *R* for pain: 0.49, *p* < 0.001; Fig. 2A), but no significant correlation for OH and PH (Spearman’s R for OH: 0.22, *p* = n.s.; for PH: 0.24, *p* = n.s.). This suggests that pain ratings of PAT and PSY closely align. MH ratings correlated significantly, and PAT quantified their MH only slightly lower with no statistical significance than PSY do. In contrast, assessments of OH and PH did not show any relation at all between the groups.

We next focused on possible correlations between disease perceptions by INT and PAT. The dissonant gradings of patients’ OH, PH and pain (Table 3B) fully translated onto the correlation matrix, revealing no statistically significant correlation at all for any of the four health domains assessed (Spearman’s *R* for OH: 0.13, *p* = n.s.; for MH: 0.27, *p* = n.s.; for PH: 0.18, *p* = n.s.; for pain: 0.27, *p* = n.s.; Fig. 2A). These findings indicate that the assessments of a PC patients’ condition may completely diverge between PAT and INT.

Contrary to the discrepancies between PAT and PSY/INT, assessments of INT and PSY were more aligned: The correlation analysis showed a positive correlation with respect to the rating of all four health domains (Spearman’s *R* for OH: 0.41, *p* = 0.002; for MH: 0.46, *p* < 0.001; for PH: 0.34, *p* = 0.012; for pain: 0.39, *p* = 0.004; Fig. 2A). Taken together with the data from Table 3C, this indicates that INT graded patients’ OH and PH as less impaired and pain as less severe than PSY, while a positive correlation between the two observer groups was still present. MH ratings of INT and PSY aligned closely.

Of note, within the patients’ own assessments, the PAT grading of MH correlated positively with OH (Spearman’s *R* = 0.62, *p* < 0.001) and PH (Spearman’s *R* = 0.54, *p* < 0.001). However, no significant correlation between MH and pain (Spearman’s *R* = 0.11, *p* = 0.422) was found. In line with our expectations, OH and PH showed a positive (Spearman’s *R* = 0.82, *p* < 0.001), OH and pain as well as PH and pain a negative correlation (Spearman’s R for both = − 0.34, *p* = 0.011 and *p* = 0.012, respectively). This translates into OH, MH and PH all depending on each other, while OH and PH were perceived as better at low levels or in the absence of pain. Interestingly, pain and MH did not seem to depend on each other in our cohort of patients.

### Disease perceptions by PAT, INT and PSY and possible correlations with other PROMs and objective findings

Next, we assessed possible correlations of disease perceptions with other PROMs and objectifiable findings (Fig. 2B). The strongest and/or most numerous correlations of health ratings were found with the psychological and physical health section of the WHOQoL-BREF, PHQ-9, FSS and Karnofsky index.

The psychological health section of the WHOQoL-BREF correlated well with the patients’ own perception of OH, MH and PH, but not pain (Spearman’s *R* for OH: 0.60, *p* < 0.001; for MH: 0.75, *p* < 0.001; for PH: 0.41, *p* = 0.002; for pain: 0.25, *p* = 0.066; Fig. 2B). It showed a decently positive correlation with PSY assessments of MH (Spearman’s *R*: 0.48, *p* < 0.001), but no statistically significant correlations with any other assessments by PSY or INT.

Similarly, the physical health section of the WHOQoL-BREF correlated positively with the patients’ own perception of OH, MH and PH and negatively with pain (Spearman’s *R* for OH: 0.60; for MH: 0.46; for PH: 0.54; for pain: − 0.66; all *p* values < 0.001; Fig. 2B). It showed statistically significant positive correlations with INT assessments of OH and MH (Spearman’s *R* for OH: 0.30, *p* = 0.034; for MH: 0.29, *p* = 0.046) and a negative correlation with PSY assessments of pain (Spearman’s *R*: − 0.35, *p* = 0.014), but no statistically significant correlations with any other assessments by PSY or INT.

The PHQ-9 score correlated negatively with the patients’ perception of their OH, MH and PH, and positively with pain (Spearman’s *R* for OH: − 0.50, *p* < 0.001; for MH: − 0.58, *p* < 0.001; for PH: − 0.45, *p* < 0.001, for pain: 0.30, *p* = 0.029; Fig. 2B). It also correlated negatively with PSY assessments of MH (Spearman’s *R*: − 0.33, *p* = 0.023), but no significant correlations were found regarding the remaining assessments by PSY or INT.

Just as the PHQ-9 score, albeit slightly less pronounced, the FSS correlated negatively with the patients’ perception of OH, MH and PH, and positively with pain (Spearman’s *R* for OH: − 0.41, *p* = 0.002; for MH: − 0.45, *p* < 0.001; for PH: − 0.30, *p* = 0.026, for pain: 0.40, *p* = 0.002; Fig. 2B). No statistically significant correlations were found with any of the assessments by PSY or INT.

Lastly, the Karnofsky index correlated well with patients’ perceptions of OH, MH and PH, but not pain (Spearman’s *R* for OH: 0.40, *p* = 0.004; for MH: 0.39, *p* = 0.005; for PH: 0.36, *p* = 0.010, for pain: 0.23, *p* = 0.121; Fig. 2B). Further, positive correlations were found between the Karnofsky index and INT assessments of OH and MH (Spearman’s *R* for OH: 0.48; for MH: 0.54; both *p* values < 0.001) and PSY assessments of MH (Spearman’s *R*: 0.28, *p* = 0.038). Of note, the Karnofsky index did not correlate with pain ratings of any of the groups.

Interestingly, none of the health assessments by any of the groups correlated with the overall amount of diagnostic procedures performed, not even with the number of pathological findings in diagnostic procedures (Fig. 2B). Few and/or rather weak correlations with statistical significance were found for the environment and social relationship section of the WHOQoL-BREF, working status, number of medical contacts, somatic or psychiatric diagnoses.

## Discussion

Our data indicate that the perception of a PC patient’s state of health may completely differ between them and their attending health care professionals. Strikingly, we found no relevant correlations of statistical significance between PAT and INT in any of the four health domains assessed. Further, PAT grade their OH, PH and their pain as more severe than INT do. Taken together, this suggests that the assessment of a patient’s condition may completely diverge between PAT and INT. Similarly, when comparing disease perceptions by PAT and PSY, the assessments of OH and PH did not correlate well between the two groups and both health domains were perceived as more severely impaired by patients. In contrast, MH and particularly pain ratings of PAT and PSY seemed to closely align.

Interestingly, we also found some dissonance in the perceptions of patients’ health between INT and PSY: INT graded patients’ OH and PH as less impaired and pain as less severe than PSY, while a positive correlation between the two groups was detected. In contrast, MH appraisal did align very closely between those two groups.

Our findings have crucial implications for the clinical management of PC patients. The very high prevalence of the PC condition poses significant pressure on health care systems around the world, often testing the patients’ trust in health institutions by itself. Different perceptions of the patients’ health by their treating INT and/or PSY may raise trust issues and compromise the patient-physician relationship. Thus, it is of central importance to understand the underlying causes for the dissonant assessments and which conclusions to draw thereof. To this end, we closer investigated potential causes for the diverging disease perceptions and tried to identify assessments and parameters that could provide common ground for even communication between patients and health care providers.

One possible explanation for the dissonant disease perceptions between PAT, INT and PSY may lie in different experiences regarding health and disease. Individuals with professions outside the medical field commonly have less frequent exposure to severely ill patients. It seems fair to assume that our cohort of relatively young PC patients (Mdn age of 39 years), most of whom have had non-severe courses of COVID-19, may never or just rarely have experienced critical disease by themselves or in their social environment. Physicians, on the other hand, are frequently exposed to critically ill patients (i.e. in the context of intensive, intermediate or palliative care settings). This may shape their individual grading of health categories differently. Different patient collectives and clinical entities might also explain the differences when comparing INT and PSY health assessments. Taken together, personal experiences regarding health and disease may account for different interpretations of both the Likert scales and the NPRS, explaining some of the discrepancies between medians of health ratings by different observers. However, this fails to explain the many missing correlations between disease perceptions between PAT and INT/PSY, which should be present if only the Likert scales and NPRS were interpreted differently.

Our data demonstrate that patients grade all facets of their health, including pain, as worse if they show signs of mood disorders (WHOQoL-BREF – psychological health section) such as depression (PHQ-9), or fatigue (FSS). Depressive disorders have been described as more frequent in PC patients relative to survivors of infections caused by different pathogens and show a high prevalence in PC patients [1, 15, 16]. This is reflected in a high median PHQ-9 score of our PC patient cohort. The cognitive model of depression lays out several examples of cognitive distortions [17, 18], of which catastrophizing and polarizing may explain why patients suffering from a depressive state would rate many aspects of their health considerably worse than INT or PSY.

Interestingly, we found none to very few correlations between disease perceptions and objectifiable findings (e.g. number of pathological findings in diagnostic procedures, number of medical contacts or pre-existing somatic/psychological conditions, etc.). The lack of convincing correlations between disease perceptions and objectifiable findings at first appears counter-intuitive. It should be stated (restrictively) that at the time of writing, no biomarkers have yet been identified to objectively confirm the presence of a PC condition. Most abnormal diagnostic findings were of subordinate clinical relevance (e.g. lack of vitamin D or folic acid, hypercholesterinemia; data not shown) and unlikely to cause or significantly contribute to the patients symptoms. Consequently, the number of pathological findings correlated strongly with the number of diagnostic procedures performed but did not translate into high numbers of relevant findings likely to account for the patients’ complaints.

The lack of correlations between perceived morbidity and objectifiable findings, the mostly diverging disease perceptions between PAT and healthcare professionals, as well as inverse correlations found between patients’ health gradings and mood disorders are indicative of functional complaints and confounding psychosomatic factors modulating the patients’ disease perceptions. Importantly, while this may not be true for every individual patient of our cohort, the effect appears pronounced enough to pertain to a significant portion of our patient collective. Our observations are in line with a prospective study performed on *n* = 137 patients by Milde et al. The authors identified psychosomatic symptom burden [assessed by the Somatic Symptom Disorder—B Criteria Scale (SSD-12)] and other psychological factors (i.e., chronic stress, depression) to predict higher odds and magnitude of COVID-19 related symptom impairment in up to 6 months after SARS-CoV-2 infection, as well as fear of COVID-19 related health consequences [assessed by the Fear of COVID-19 Scale (FCV-19)] to increase the odds of reporting any PC symptoms [19]. Lier et al. identified a phenotype of PC patients with the predominant symptoms fatigue, somatization and depression, whose standard laboratory and cardiopulmonary biomarkers did not differ from a control group of neuropsychiatrically unaffected PC patients [20]. Within this group of patients, fatigue, somatization and depression correlated with worse functional outcomes measured by means of the Post COVID-19 Functional Status scale (PCFS) [21]. Lastly, Alhanbali et al. failing to objectify differences in auditory impairment between PC patients with and without reported hearing difficulties using a broad range of diagnostic tools [22] corroborate the hypothesis of somatization disorders and psychosomatic causes fueling subjective morbidity in a fraction of PC patients.

Taken together, different interpretations of the Likert scales and the NPRS as well as concomitant mood disorders (i.e. depression) and psychosomatic factors may explain the diverging disease perceptions by PAT, INT and PSY. To overcome this health perception gap, health care providers should use standardized scores. The physical health section of the WHOQoL-BREF and Karnofsky index both correlated positively with OH and MH assessments by PAT and INT. They represent valid tools to estimate a PC patient’s state of health at a given time point. PROMs such as Likert scales addressing various health domains and the NPRS remain valuable tools to monitor patients’ subjective recovery or symptom aggravation over time. However, findings by different observers from different timepoints cannot be easily compared and should not be used to monitor course and outcome of PC patients. If a patients’ disease perception strongly differs from the assessments by his/her attending physician or psychologist, screening tools for mood disorders such as the PHQ-9 or the psychological health section of the WHOQoL-BREF should be applied. Further, it is advisable to evaluate possible psychosomatic symptoms by means of e.g. the SSD-12 and FCV-19 [19].

It is important to state that the presence of psychological or psychosomatic symptoms does not rule out other causes for a PC patient’s complaints. Psychological impairment may develop as a secondary condition due to persisting somatic symptoms [23, 24] and thus, PC patients’ complaints should always be taken seriously. Up to date, the extent to which the health perception gap may affect treatment and prognosis of PC patients remains elusive. To this end, we strongly encourage further studies involving long-term patient follow-up. Due to the heterogenous nature of the PC condition, it remains imperative to assess PC patients in an interdisciplinary clinical fashion.

## Data Availability

All clinical data will be made available upon reasonable request.
